# Comparison of Different Technologies for Soft Robotics Grippers

**DOI:** 10.3390/s21093253

**Published:** 2021-05-08

**Authors:** Silvia Terrile, Miguel Argüelles, Antonio Barrientos

**Affiliations:** Centre for Automation and Robotics (CAR), Universidad Politécnica de Madrid—Consejo Superior de Investigaciones Científicas, 28006 Madrid, Spain; miguel.arguellesho@alumnos.upm.es (M.A.); antonio.barrientos@upm.es (A.B.)

**Keywords:** grasping and manipulation of soft objects, soft robotics, soft gripper, pneumatic gripper, jamming gripper, passive gripper

## Abstract

Soft grippers have experienced a growing interest due to their considerable flexibility that allows them to grasp a variety of objects, in contrast to hard grippers, which are designed for a specific item. One of their most remarkable characteristics is the ability to manipulate soft objects without damaging them. This, together with their wide range of applications and the use of novels materials and technologies, renders them a very robust device. In this paper, we present a comparison of different technologies for soft robotics grippers. We fabricated and tested four grippers. Two use pneumatic actuation (the gripper with chambered fingers and the jamming gripper), while the other two employ electromechanical actuation (the tendon driver gripper and the gripper with passive structure). For the experiments, a group of twelve objects with different mechanical and geometrical properties have been selected. Furthermore, we analyzed the effect of the environmental conditions on the grippers, by testing each object in three different environments: normal, humid, and dusty. The aim of this comparative study is to show the different performances of different grippers tested under the same conditions. Our findings indicate that we can highlight that the mechanical gripper with a passive structure shows greater robustness.

## 1. Introduction

Robotics grippers are one of the most important components in the industry because of their capability to manipulate objects. They not only realize transportation tasks but also perform quality control to optimize robotic work cells [1]. They can be used in several applications, such as industry, medicine, space exploration, and so on. Each application presents different characteristics and needs specific solutions, so grippers can be divided into categories based on their function as described in [2]. Thanks to the advance in soft material and soft actuators, soft grippers have gained relief in the field of soft robotics in the last years.

Traditional hard robotic grippers consist of a set of mostly rigid joints and links [3] that have to precisely adapt to the object to not damage it. Consequently, their use is limited to a specific item type. If a variety of objects needed to be manipulated, it will be very difficult, if not impossible, to grasp them with the same rigid gripper.

The solution is to increase the degrees of freedom of the fingers that form the gripper, using, e.g., flexible materials for finger fabrication. This allows grippers to passively adapt to objects. Using the principles of soft robotics, gripping tools with a greater robustness can be developed. This implies that they do not require changes in their programming or construction to manipulate different objects, and the precision needed to make the grips is reduced.

## 2. Related Work

Although the development of soft robotics is relatively recent, a variety of soft grippers exist, as seen in [4].

Rigid gripping tools with electromechanical actuation are the first step toward soft robotics. Using a chain of rigid elements, grippers can adapt to the shape of the objects. The greater the number of elements that compose the chain, the better the finger profile approximate to that of a continuous line. This is interesting because a larger contact area between the gripping tool and the object leads to a reduced pressure exerted on that area, thus resulting in a lower risk of damaging delicate objects. A prototype based on this idea was developed in 1978. This gripper could adapt to objects of almost any shape and size and grip them with uniform pressure along with the whole finger. Moreover, it presented a relatively simple mechanism and control implemented only with the traction of a pair of wires [5].

An evolution of the previous example is a tendon-driver gripper fabricated through shape deposition manufacturing, which presents joints formed by an elastomer as well as actuators and sensors incorporated in hard rigid polymers [6]. In another case, two different types of silicone have been used to realize the fingers of the gripper: one more rigid for the base and another more flexible for the section to be in contact with objects. The actuation is realized only with a cable to allow an easier adaptation of the gripper to objects [7]. Other examples are [8,9,10,11,12].

The need to increase the contact surface between the finger and the object is also the idea behind the grippers that use passive structures actuated with external motors. In this case, cables are not required, because when grippers deform, they adapt to the geometry of the objects. The fourth gripper presented in this study and in [13] are based on this concept.

Another large family of soft grippers is the one using pneumatic actuators. In general, they have deformable pneumatic chambers realized with different types of silicone or other elastomers. What distinguishes them is the shapes of the pneumatic chambers. Examples of this type of gripper can be seen in [14,15,16,17,18,19].

Two interesting types of pneumatic soft grippers are those that use the pneumatic system to create the vacuum and grasp objects by suction, such as [20,21], and those that exploit the principle of jamming transition such as [22,23,24,25]. It is also worth mentioning the fabric-based soft grippers; these pneumatic actuators present some advantages over others due to their high flexibility, stiffness customizability, and high force-to-weight ratio [26,27,28].

Apart from these two categories into which the four grippers described in this research fall, many other technologies have been used: electro-adhesion [29,30,31], Gecko-adhesion [32,33], dielectric elastomer actuators (DEAs) [34,35,36,37], fluidic elastomer actuators (FEAs) [38,39], magnetorheological (MR) fluid [40], and shape-memory materials [41]. In some cases, two different types of actuation are implemented in the same gripper to allow stable and reliable grasping under different working environments as in [42], which presents the combination of the suction and traditional linkage-driven grippers or in [18], which presents the integration of an electro-adhesive system and a pneumatic actuator.

All these last technologies have achieved good results, although to date, they are not yet used, especially in the industrial sector, since they do not have the same performance as pneumatic and mechanical grippers.

This work presents a review and comparison of different technologies for soft robotic grippers to highlight the more robust mechanical design. Usually, specific robotic gripper designs are presented and tested to demonstrate their effectiveness. Nevertheless, soft robotic grippers present the important characteristic of gripping a variety of objects, so it is useful to know what the best option is and why when robustness is the first requirement. Our results have the purpose of showing how different types of mechanical actuation systems and material selection can influence gripping capability.

The mechanical design of grippers is illustrated in Section 3, describing separately the first two pneumatic grippers and the last two mechanical grippers. Then, experiments and results are discussed in Section 4, followed by the conclusions in Section 5.

## 3. Mechanical Design of Grippers

In this work, four soft robotic grippers have been recreated starting from commercial products, except for the third one. Each gripper has been designed from scratch, thus being able to modify in part their original design, but always aiming for the finest quality. To realize an effective comparison, two actuation systems (pneumatic and electromechanical) and four design solutions have been chosen. More specifically, two grippers employ a pneumatic actuation system, while the another two utilize an electromechanical actuation system. In addition, grippers present similar sizes and fit the UR3, which is the robotic arm used in the experiments.

In the first part of this section, each gripper will be described in terms of its design and actuation. The two type of actuation systems are separately described, being common to every pair of grippers.

### 3.1. Pneumatic System

**Figure 1:**
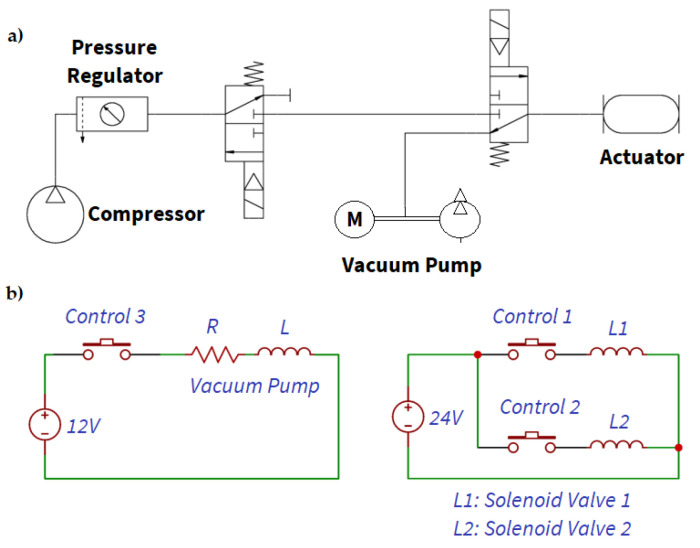
Pneumatic system. (**a**) Connection diagram. (**b**) Wiring diagram control. Signals 1, 2, and 3 are the cables that connect to the UR3 for control.

The two pneumatic grippers use the actuation system shown in Figure 1.

The pneumatic module is composed of two solenoid valves. One of the solenoid valves is used to connect the path that carries pressurized air to the gripper with the one from which air is extracted with the vacuum pump. The other solenoid valve connects the tube from the pressurized air reservoir with the inlet of the first solenoid valve and a blind tube. It operates as an open/close valve. Finally, a pressure regulator controls the pressure that is administered to the gripping tools. For compressed air, a compressor with a small tank is used, whereas an electric vacuum pump is used for vacuum generation.

#### 3.1.1. Gripper 1

**Figure 2:**
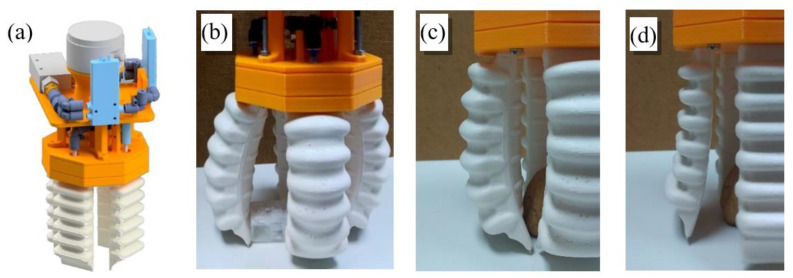
First gripper: (**a**) CAD model, (**b**) Gripper during the operation, (**c**) Fingers flexed, (**d**) and Fingers in extension.

The first gripper, shown in Figure 2, is a pneumatic gripper based on the one commercialized by Soft Robotics [43]. They developed several configuration solutions depending on the application. In this work, the four-finger solution, with fingers symmetrically spaced around the center of the gripper, has been adopted.

The most important aspect of the design of fingers is its asymmetry along the lengthwise plane. This is necessary to allow movements. The inner side presents a flat surface with small reliefs that stick out to improve gripping capacity. The outer side presents six chambers that inflate when pressurized air is introduced inside, generating the contraction movement. Vice versa, creating a vacuum inside, they contract and generate the extension movement.

At one end, the finger has a section that allows it to be held at the base of the gripper, as well as allowing air to enter and exit the chamber. On the other end, a section with greater rigidity and with a triangular profile has been added to the finger. This provides a relatively straight section at the end of the finger to perform a better grip with the extremity. Fingers measure 11 cm in length and 4.5 cm in width.

For the creation of this prototype, two parts can be distinguished: manufacturing silicone fingers and the 3D printing of the rest of the pieces that form the base of the gripping tool (all 3D printed parts are made with the thermoplastic polyester PLA).

**Figure 3:**
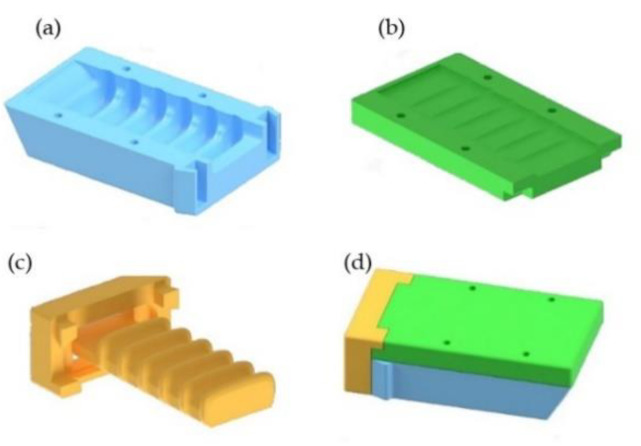
Mold for manufacturing silicone fingers: (**a**) Mold for inner side, (**b**) Mold for outer side, (**c**) Male mold, (**d**) The assembled mold.

To obtain a silicone finger, a mold has been realized with 3D printing, as shown in Figure 3. It comprises three different parts: a male that provides the internal shape of the finger, and two females, which provide the exterior. The silicone used is the Silastic^®^ 3483, with a cure time of one hour.

This silicone allows getting the desired characteristics with regard to stiffness, while other silicones such as Ecoflex^®^ 00-30 have proved too soft for this application.

The working pressure of the gripping tools is around 2 bars.

#### 3.1.2. Gripper 2

**Figure 4:**
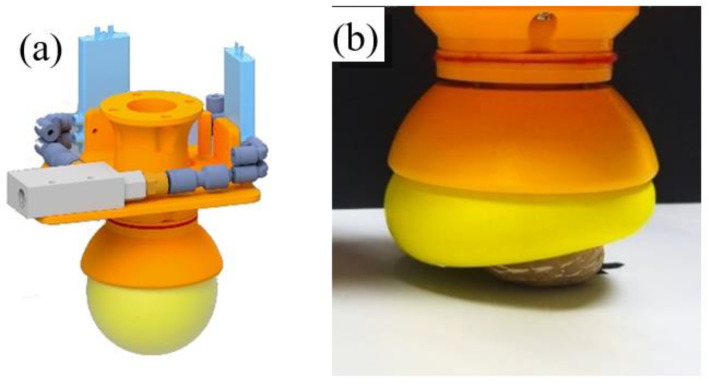
Second gripper: (**a**) CAD model of the gripper, (**b**) Gripper during the operation.

The second gripper, as shown in Figure 4, is also pneumatic and is based on the Versaball, which is a gripper produced by the company Empire Robotics Inc. [44]

Its operation is based on the jamming process by which granular materials increase their viscosity when the density of particles increases.

The gripper presents a spherical chamber made of an elastomeric plastic membrane filled with granular material. It is possible to get the liquid and solid pseudo-states when pressurized air and vacuum are applied, respectively.

To grab an object, a small amount of air has to be introduced in the chamber so that the granular material acts similar to a liquid. Then, the gripper has to be pressed against the object, adapting the contents of the chambers to the outer shape of the object. Finally, the vacuum is created inside the chamber, in order to solidify its interior and maintain the shape of the object. As long as the vacuum is maintained, the object is held by the gripper. Subsequently, it is enough to fill the chamber with air again, to return to the “liquid” state and release the object. This method of gripping requires that the object size be approximately half of the diameter of the pneumatic chamber.

This gripper is composed of the previously described pneumatic module, a piece to join this module with the claw, two pieces to keep the filter in place, and two final pieces that hold the pneumatic camera.

As in the previous case, the pneumatic chamber is the basic element of the gripper. To realize it, a latex balloon of approximately 1-mm thickness and about 8 cm in diameter has been chosen. The chosen thickness provides the chamber with resistance against the abrasive action of the granular material and ensures that it is not damaged when coming into contact with objects. At the same time, a smaller thickness adapts easier to the small irregularities of the objects, besides gaining flexibility and elasticity.

Several options of granular materials have been tested, such as ground coffee, fine sand, and breadcrumbs. After testing ground coffee and breadcrumbs, it has experimentally been observed that with ground coffee, the chamber adapts much better to objects. This is the same material used in the research previously mentioned.

A filter is necessary to allow air to pass in both directions and maintain the granular material isolated from the pneumatic system. The filter is composed of a felt circle with the same diameter as the upper section of the base and a thickness of approximately one millimeter. The working pressure is around 1 bar.

### 3.2. Electromechanical System

The other two grippers are mechanical and use a 6 V DC motor with a reduction gearbox. To calculate the reduction, two parameters must be considered: the approximate closing time of the gripper and its generated torque.

For the third gripper, a great reduction ratio is necessary, since the torque generated by the motor must be enough to pull the three cables of the three fingers of the gripper simultaneously, so a reduction of 1:298 is necessary at least.

Indeed, a fourth gripper presents a spindle nut system that generates a suitable torque for this application. Thus, the reduction is chosen only based on the gripper closing time and is equal to 1:125.

The same structure is used to mount the motors on the two grippers, as the motor size is identical in spite of the different reduction ratio. This structure is composed of two parts: a base and an adapter for the robotic arm UR3. The base part houses the motor inside and is designed to be tightened to prevent its longitudinal displacement.

**Figure 5:**
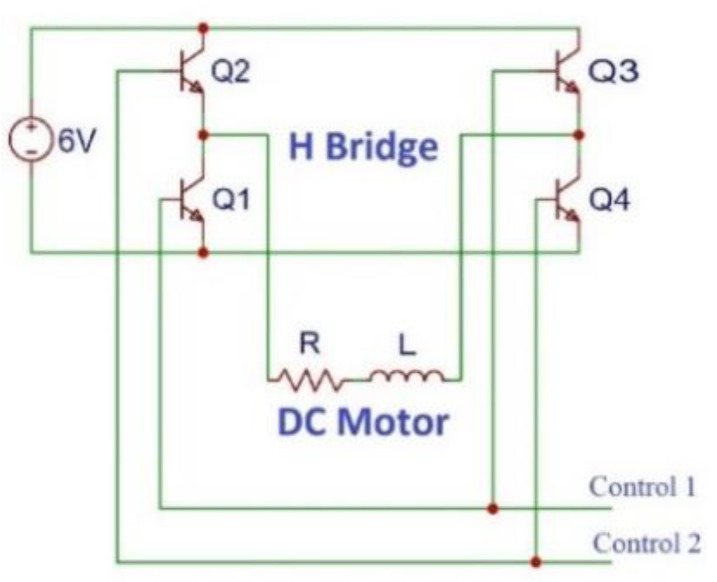
Wiring diagram of the electromechanical system.

Finally, the simplified electrical scheme is showed in Figure 5. The LR group represents the DC motor, and the H bridge is composed of the four bipolar signals and the two control signals. These are the ones that connect to the input/output panel of the UR3.

#### 3.2.1. Gripper 3

**Figure 6:**
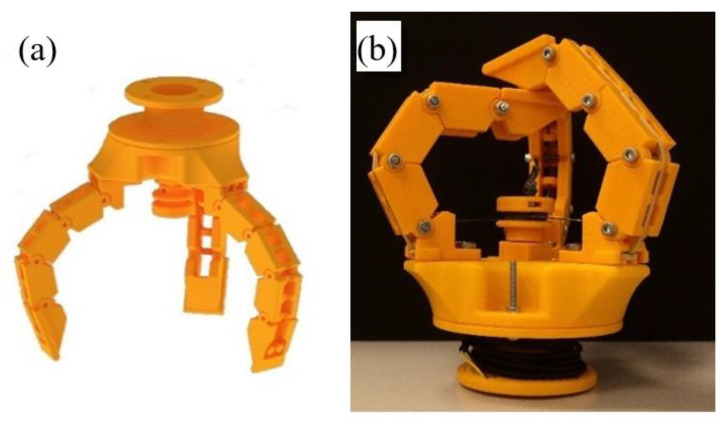
Third gripper: (**a**) CAD model of the gripper, (**b**) Real Gripper.

The first mechanical gripper, as shown in Figure 6, presents articulated fingers actuated by cables. When the cables are pulled by a motor connected to a pulley, the fingers are flexed.

The shape that fingers adopt is a function of the object to be grasped; the links of the finger will adapt to the outer surface of the object.

Here, the number of fingers selected to achieve optimum grip is three, which are arranged at 120 degrees. To work properly, the three fingers must perform their movements simultaneously.

To carry out this movement, the three cables (one for each finger) are wound on the same pulley that collects or releases the cables, generating thus the flexion or extension of the fingers on the basis of the rotating direction.

The pulley represents a crucial part of the design, since it is responsible for the synchronization of the movement of fingers. It has an inner section in which a nut is inserted to fasten the pulley to the flat face of the motor shaft. The pulley is designed with a diameter that is small enough to generate a good torque for the application but large enough to be printed in 3D and not too fragile. It also has a duct with a circular section that connects the outer face of the pulley, where the cables are wound, with the underside of the pulley.

The three fingers are held on a base, and an adapter is used for the connection to the robot. Each finger comprises three base modules, one end, and a mount. Modules are joined with screws that allow their relative rotation. Each finger measures 12 cm in length and 2.5 cm in width.

#### 3.2.2. Gripper 4

**Figure 7:**
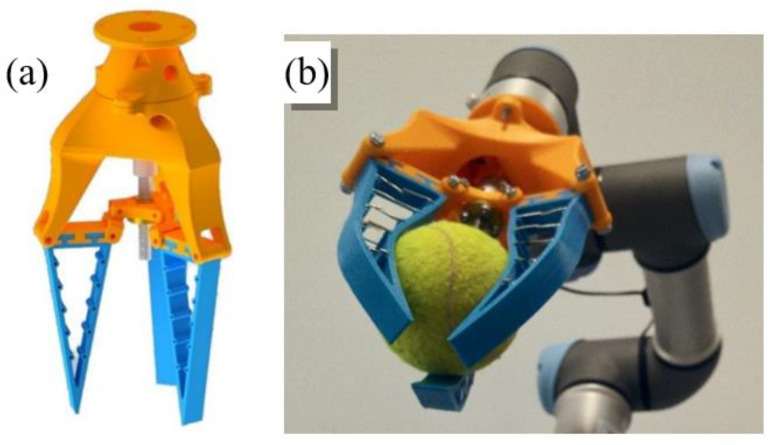
Fourth gripper: (**a**) CAD model of the gripper, (**b**) Gripper during the operation.

The fourth gripper, as shown in Figure 7, is based on the MultiChoice Gripper product manufactured by the FESTO^®^ Company [45]. This design is based on the mechanics of the human hand where the thumb serves to impose an effort in the same direction but opposite orientation as the rest of the fingers. To adapt to different object typologies, the tool developed by FESTO is able to reorient the fingers and obtain different configurations. For example, for objects with round perimeter are placed with ternary symmetry. However, for objects with parallel faces, one finger is placed in opposition to the other two.

Nevertheless, for the reproduction of this prototype, this feature has been obviated to focus attention on the design of the fingers: passive structures capable of adapting to the objects that deform them. This gripping tool comprises three fingers of a flexible material. The fingers are triangular with thin walls and, longitudinally, they present a nucleus formed by rigid parts that prevent the walls of the finger from approaching. With this morphology, the tip remains approximately in the same place when trying to deform the finger, but the body bends around the object. This deformation of the finger allows it to adapt to the shape of the surface of the object with which it will make contact, and, thanks to its flexibility, it will deform before damaging it. In this case, the choice of the number of fingers that form the gripper could have been two, three, or four. The number three has been selected because the spindle mechanism makes the movement perfectly synchronized, and it is unnecessary to complicate the design by adding a fourth finger, although in some scenarios, it might be useful to have it.

The gripping tool consists of three flexible fingers and a rigid base. The fingers are made from the elastomeric thermoplastic produced by the company Recreus [46] called FilaFlex, and they measure 11 cm in length and 1.5 cm in width. The base is formed by two pieces: the main one on which the fingers are mounted and to which the motor is coupled, and a secondary one that serves to join the gripping tool with the UR3. Furthermore, the mechanical actuation is composed of a direct current motor, a coupler flexible, a spindle and a nut, and a handle to which the nut, the finger connector as well as the motor support are attached.

To generate the grip movement, each finger can be held with rotational joints at both ends of its base. By maintaining the position of the outer ends of the finger bases and changing the height of the inner ends, the opening and closing movement of the hand is achieved. This movement, which can be generated in many ways, is done with a spindle nut mechanism. A motor rotates the spindle, which causes the nut to move up and down when its rotation is prevented. The inner ends of the fingers are connected to the nut by connectors, which allow the possibility of movement while preventing the rotation of the nut.

The rigid sections that form the internal structure of the finger have been made with a wire. This wire has enough rigidity to allow the deformation of the finger but at the same time allow it to return to its initial position.

## 4. Experiments

Preparation of the Experiments

**Figure 8:**
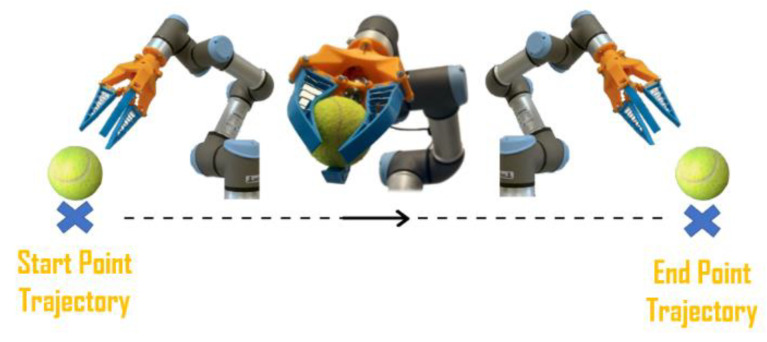
Test execution: the gripper picks up one of the twelve objects in a predetermined position and place it in the desired location.

The objective of this work is to test the robustness of the gripping tools and their grasp stability. To test these parameters, an experiment has been designed in which the ability of the gripper to grip a certain object, to hold it while the robotic arm realizes a trajectory, and successively release it, is evaluated (as illustrated in Figure 8). The test is deemed successful if the object does not fall either during the grip or the displacement and if the object is not damaged after gripping action.

**Figure 9:**
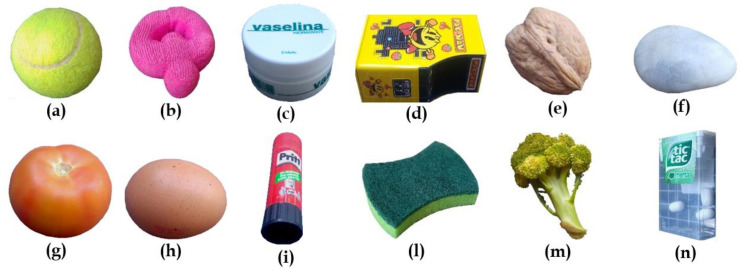
Selection of objects for experimentation with its identification number. (**a**) Tennis ball #1, (**b**) Screen Cleaner #2, (**c**) Vaseline Container #3, (**d**) Metallic Container #4, (**e**) Nut #5, (**f**) Rock #6, (**g**) Tomato #7, (**h**) Egg #8, (**i**) Glue Stick #9, (**l**) Dish Sponge #10, (**m**) Broccoli #11, and (**n**) Candy Box #12.

Since one of the most important aspects is the capability of grippers to manipulate objects with different characteristics, i.e., different shape, rigidity, and fragility, twelve objects, as shown in Figure 9, have been chosen for experimentation. Each of them presents different characteristics to represent a wider range of objects. The weight and dimensions of objects are limited by the capacity and dimensions of the grippers.

**Figure 10:**
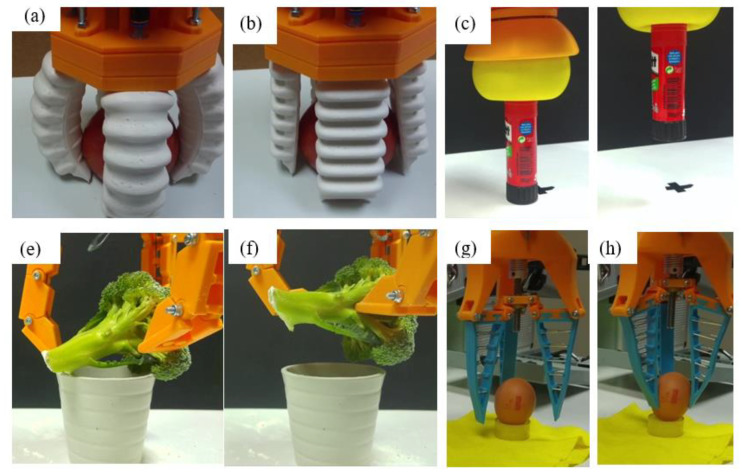
Test execution with Gripper 1 (**a**,**b**), Gripper 2 (**c**,**d**), Gripper 3 (**e**,**f**), Gripper 4 (**g**,**h**).

Objects are oriented in different ways to not facilitate any gripper. For example, object #9 (see Table 1) is arranged vertically, whereas object #12 is arranged horizontally. In some cases, external elements have been used to ensure the correct positioning of the object, such as for the egg (#8) (as can be seen in Figure 10 or for the tennis ball (#1), which would have slipped once placed on the surface due to their physical characteristics.

In the following table, an identification number is assigned to each object to simplify the result tables.

The experiment has been carried out in three different environments: normal, humid, and dusty. The normal environment is achieved without changing the properties of the environment. In this case, objects are not damp or dusty.

The humid environment consists in moistening both the gripping tool and the object. The presence of humidity causes greater effects on some objects than on others, depending on the surface of the object and how much the water can wet it. For instance, while a plastic container with smooth walls may only slightly be affected by a humid environment, properties of a sponge, such as its weight and surface adhesion, greatly change in such an environment. Finally, the dusty environment is simulated by spreading a generous amount of flour both by the tool and by the object. The last two situations can occur in agriculture or in specific industrial processes.

For each experiment, 20 repetitions have been performed. This means that for each object, 60 repetitions have been performed with each tool and twenty have been performed for each environment, which are 240 for each gripper and object. Being twelve objects, a total of 2880 repetitions are performed. In Figure 10, it is possible to see some captions of these experiments.

The time used for successful gripping is different for each of the grippers. In the case of Gripper 1, this time is in the range of 1–2 s, since the fingers must be inflated to a greater or lesser extent according to the object’s size. In the case of Gripper 2, this time is about 3 s, since it is necessary that the gripper first adjusts to the shape of the object to subsequently perform the vacuum. In the case of Gripper 3 and Gripper 4, this time is less than or equal to one second.

## 5. Discussion

The data obtained from the experiments are presented in the following graphics and Table 2, Table 3, Table 4 and Table 5.

The first three columns of data correspond to the success rate of the gripping tools in the three scenarios: normal, humid, and dust. The average column of success rates for each object in all scenarios is presented in the fourth column. In the last row, the average success rate for each environment is calculated, and the global success rate is calculated as the average between the rates of each object in each scenario. Each table is accompanied by a histogram to display the data more intuitively.

**Figure 11:**
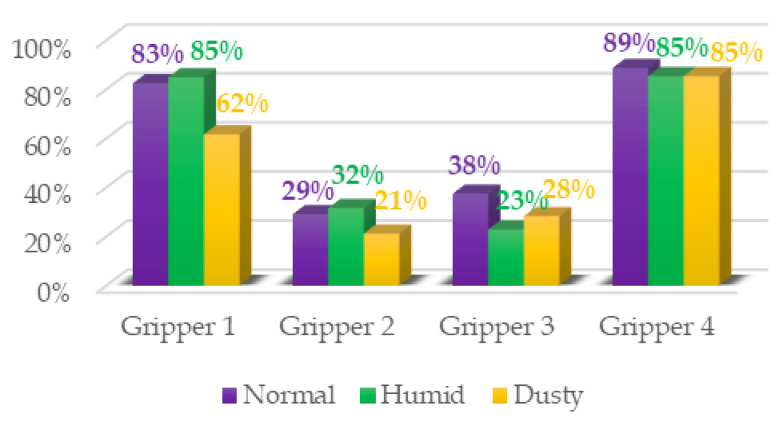
Summary of test results for the four grippers in the three environments.

From the results (Figure 11), it can be seen that the success order of the grippers is as follows: Gripper 4, Gripper 1, Gripper 3, and Gripper 2.

Gripper 1, with 77% of the global success rate, is the best of the two pneumatic grippers and ranks second among the four presented grippers. Table 2 indicates that Gripper 1 can grab eight of the 12 objects with a success rate equal to or greater than 85% and that only in two cases, with objects #6 and #8, it has not been able to grasp them. This is because the size of object #6 (the stone) was too small for this type of gripper, while the surface of the object #8 (the egg) was the most slippery, besides the impossibility of applying high forces to avoid breaking the object. The best performance is achieved in a humid environment. This is because the silicone of the fingers has increased adhesion in the presence of moisture. Vice versa, the friction coefficient decreases in the presence of dust, and therefore, the worst results are obtained in the third scenario.

Gripper 2 presents the lower global success rate, which is equal to 27%. This was expected due to the restriction by which it cannot take objects more than 50% of its diameter. It achieved good results only with the smallest and lightest objects, but also, in this case, it did not do better than the other grippers. For the same reasons as in the previous case, the presence of humidity seems to indicate better performance.

Gripper 3, with 30% of the global success rate, is slightly better than Gripper 2. However, it is evident from Table 4 that it is not better. Despite its ability to grab many of the objects, it fails to complete the task in most cases. It reached 100% only in three cases: with object #2 in the normal environment and with object #7 in the normal and dusty environment. These results indicate that this gripper is suitable for large objects and is not affected by weight. Unlike Gripper 2, it is unable to grasp small or rectangular objects, and the presence of humidity, in general, worsens its performance (as in the case of objects #2, #7, and #10).

Gripper 4, with 86% of the global success rate, is the best gripper of this study. As can be seen in Table 5, its performances, with a success rate of 89% in the normal environment and of 85% in the other two cases, are not significantly influenced by the type of environment. It is capable of grasping both small and light objects as large and heavy objects. Fingers adapt to objects by applying uniform pressure without the need to exert much force. The gripper presents problems only with two objects, #4 and especially #12. In both cases, it is a rectangular object with smooth surfaces (in the case of #12, the thickness is least). In this case, the gripper, with its three fingers, is unable to surround the object and adapt to it.

In summary, Gripper 1 can grab many different objects with similar performance. Its limitations proved to be the dimensions below those of the nut and the thin objects. In the case of delicate objects such as #7 and #11, the gripper has left them intact. Gripper 2 can grab little and lightweight objects, better if with regular surfaces, as in the case of objects #9 and #12. Almost certainly, some improvements in the realization of the gripper would allow achieving better results, but due to the limitations mentioned above, it would still not be the most robust gripper among the four proposed. Gripper 3 is suitable for grabbing sufficiently large and heavy round objects that cannot escape laterally. In general, it did not cause damage to the most delicate objects; however, it is necessary to generate more force on the surface of the object to ensure a firm grip. Finally, Gripper 4 proved to be the most robust gripper of the four proposed. It is able to grasp objects without exerting a significant force, so it is suitable for delicate objects, and it has been seen that neither the tomato nor the broccoli has been dented.

**Figure 12:**
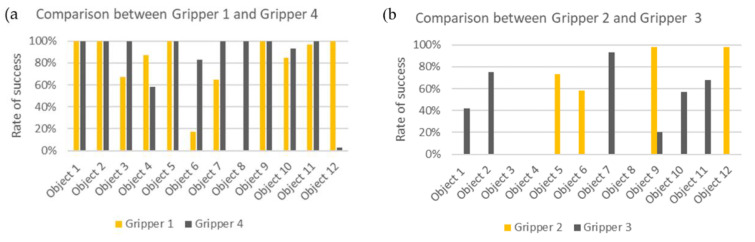

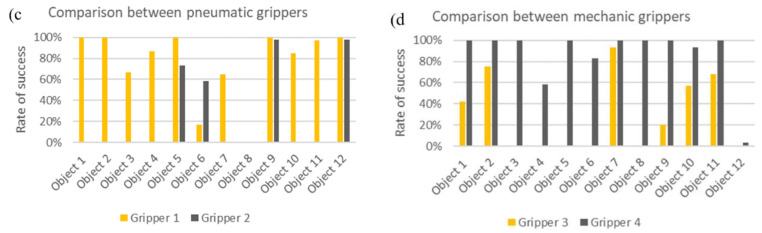
Graphic comparison between (**a**) Gripper 1 and Gripper 4. As can be seen from the graph, the two grippers generally have similar performances. (**b**) Gripper 2 and Gripper 3. These are the two grippers with the worst performances, and each one proved to be suitable only for a specific type of object. (**c**) Graphic comparison between Gripper 1 and Gripper 2 (the two pneumatic grippers). As can be seen from the graph, Gripper 1 works significantly better than Gripper 2. (**d**) Graphic comparison between Gripper 3 and Gripper 4 (the two mechanic grippers). As can be seen from the graph, Gripper 3 works significantly better than Gripper 4.

In Figure 12, comparison graphs have been realized to highlight the most significant differences.

Figure 12a compares the performance of Gripper 1 and Gripper 4, the two best grippers. Both exhibit acceptable behavior with most objects. This indicates that in general, they are very robust tools of grip. It is interesting to note that the two grippers compensate, as with objects #8 and #12, and in part with #4 and #6. Although the shape of approaching the object is similar, the presence of a fourth finger in Gripper 1 makes the difference in being able to grab rectangular objects, even if the dimensions of these same fingers are prejudicial for grasping small objects.

Figure 12b compares the two grippers with lower performances. In this case, it is evident that the type of object that each can grasp is completely different.

Figure 12c compares the pneumatic grippers. Only with object #6 is Gripper 2 better than Gripper 1.

Finally, Figure 12d compares the mechanical grippers. In this case, Gripper 4 is always better than Gripper 3. Electromechanical grippers behave best in the normal environment and not in a humid environment such as pneumatic grippers. This is because with this type of gripper, the grip of the objects depends to lesser extent on the coefficients of friction.

Analyzing the data, it is interesting to observe that for certain objects, results are not affected by the change of environment, while for others, they change significantly. Gripper 1, for example, exhibits an important fall in the success rate with tomato in the dusty environment. These significant variations can be attributed to the surface properties of the object. The surface of a tomato, e.g., is perfectly smooth and not porous. As a consequence, powder remains on its surface and directly affects the grip. This effect does not occur with the tennis ball due to its surface roughness. Another example of this phenomenon occurs with the stone. The second pneumatic grip tool, with success rates of 85% and 90% in normal and humid environments respectively, is unable to grasp the stone in the presence of dust.

Object #9 (stick glue), with an 80% success rate globally (in all environments and with all grippers), is the object exhibiting the best results. The three objects with the worst results have been objects #4 (metallic container), #6 (the stone), and #3 (the Vaseline container), with overall results of 36%, 40%, and 42% respectively.

Finally, we present some considerations with respect to the construction and operation of the grippers. The tool with the fastest and easiest manufacturing process turned out to be Gripper 4. In addition, its control is very simple and requires only a power supply, while in the case of pneumatic grippers, a compressor, a pressure air tank, and a vacuum pump are needed.

Another factor to consider is the noise generated by the tools during use. The two electromechanical grippers are much quieter than the pneumatic ones due to the work of the compressor and the vacuum pump, although the latter could be replaced by a vacuum ejector, which is much quieter.

The work environment is another factor that affects performances. In the food industry, the pneumatic tools may be a better option due to the ease and speed to clean them. Another advantage is that the compressor and the tank may be away from tools and out of the manipulation environment. In contrast, in the electromechanical tools, the operating mechanisms are in direct contact with the work environment and could compromise the safety of the installation.

If speed of action plays a paramount role in the application, as in pick and place tasks in which the speeds of opening and closing of the tools are critical for productivity, pneumatic grippers should be considered as the better option due to their higher speed.

However, from the perspective of the maintenance of gripping tools, it is not easy to determine which is the most appropriate. Although changing a component of an electromechanical gripper requires a longer time, pneumatic elements are more likely to fail than mechanic ones.

## 6. Conclusions

Four prototypes of soft robotic grippers, including their design and manufacture, have been developed for a later stage of experimentation. To realize a more effective comparison, two grippers use pneumatic system actuation, while the other two employ electromechanical actuation, and each of them shows a different solution design. All grippers have been tested with twelve objects in three different work environments. These objects have carefully been selected to be a representative group of items that could be manipulated by the grippers in a real industrial work environment. This group includes objects with different geometric and mechanical characteristics, in terms relative to the dimensions of the grippers. An overview of the work and real tests is available in Supplementary Materials.

From the results of our tests, it can be seen that Gripper 4 (the electromechanical gripper with passive structure) and Gripper 1 (the pneumatic gripper with chambered fingers) present the highest success rate, with rates of 86% and 77% respectively.

Thus, it can be concluded that Gripper 4 represents the best option, when robustness and good behavior for the manipulation of unknown objects are needed. Gripper 1 also represents an excellent option despite some limitations due to the dimensions of fingers that do not allow grasping certain types of objects.

It is worth noting that the improvements in the mechanical design of the other grippers may increase their percentage of success rate.

## Figures and Tables

**Table 1:** Selection of objects for experimentation. Each object has been assigned a number to facilitate the subsequent display of results (first column). The “Dimensions” column shows the significant dimensions of each object, and the “Weight” column shows the weight of the objects used in the experiments. In the last column “Relevant Aspects”, the aspects that differentiate each object from the others are highlighted.

ID	Object	Dimensions	Weight	Relevant Aspects
**1**	Tennis ball	70 mm diameter	57.6 g	Quite rigid and with a rough surface
**2**	Screen Cleaner	80 mm diameter 30 mm thickness	12.8 g	Considerably soft and deformable
**3**	Vaseline container	80 mm diameter 50 mm height	147 g	Very smooth surface
**4**	Metallic container	85 × 50 × 30 mm	47.9 g	Prismatic form with some irregularities, smooth surface
**5**	Nut	35 mm diameter 45 mm height	14.4 g	Very small and lightweight
**6**	Rock	25 × 15 × 10 mm	12.9 g	The smallest object of the experiment
**7**	Tomato	90 mm diameter 70 mm height	229.6 g	The heaviest object of the experiment
**8**	Egg	50 mm diameter 65 mm height	71 g	Hard but very fragile
**9**	Glue stick	25 mm diameter 100 mm height	19.6 g	Svelte with irregularities at the ends
**10**	Dish sponge	105 × 80 × 25 mm	10.2 g	Considerably soft and deformable with a slightly stiffer surface
**11**	Broccoli	90 mm diameter 120 mm height	130.4 g	Irregular object that should not be deformed
**12**	Candy box	60 × 35 × 15 mm	7.9 g	Little gripping surface

**Table 2:** Experiments with Gripper 1. On the left, the table with a success rate for every object, in the last column, the average for every object is calculated, and at the end of the table, the average success rate for every environment is determined. On the right, the graphic representation of Table 2 is reported.

***Experiments with Gripper 1***					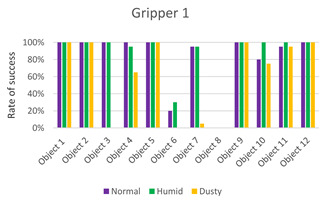
***Obj.***	**Normal**	**Humid**	**Dusty**	**Average**	
1	100%	100%	100%	100%	
2	100%	100%	100%	100%	
3	100%	100%	0%	67%	
4	100%	95%	65%	87%	
5	100%	100%	100%	100%	
6	20%	30%	0%	17%	
7	95%	95%	5%	65%	
8	0%	0%	0%	0%	
9	100%	100%	100%	100%	
10	80%	100%	75%	85%	
11	95%	100%	95%	97%	
12	100%	100%	100%	100%	
**Avg.**	**83%**	**85%**	**62%**	**77%**	

**Table 3:** Experiments with Gripper 2. On the left, the table with success rate for every object, in the last column, the average for every object is calculated, and at the end of the table, the average success rate for every environment is determined. On the right, the graphic representation of Table 3 is reported.

***Experiments with Gripper 2***					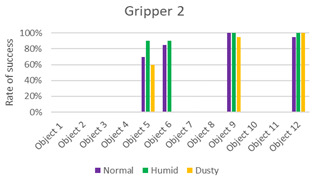
***Obj.***	**Normal**	**Humid**	**Dusty**	**Average**	
1	0%	0%	0%	0%	
2	0%	0%	0%	0%	
3	0%	0%	0%	0%	
4	0%	0%	0%	0%	
5	70%	90%	60%	73%	
6	85%	90%	0%	58%	
7	0%	0%	0%	0%	
8	0%	0%	0%	0%	
9	100%	100%	95%	98%	
10	0%	0%	0%	0%	
11	0%	0%	0%	0%	
12	95%	100%	100%	98%	
**Avg.**	**29%**	**32%**	**21%**	**27%**	

**Table 4:** Experiments with Gripper 3. On the left, the table with success rate for every object, in the last column, the average for every object is calculated, and at the end of the table, the average success rate for every environment is determined. On the right, the graphic representation of Table 4 is reported.

***Experiments with Gripper 3***					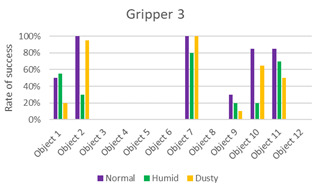
***Obj.***	**Normal**	**Humid**	**Dusty**	**Average**	
1	50%	55%	20%	42%	
2	100%	30%	95%	75%	
3	0%	0%	0%	0%	
4	0%	0%	0%	0%	
5	0%	0%	0%	0%	
6	0%	0%	0%	0%	
7	100%	80%	100%	93%	
8	0%	0%	0%	0%	
9	30%	20%	10%	20%	
10	85%	20%	65%	57%	
11	85%	70%	50%	68%	
12	0%	0%	0%	0%	
**Avg.**	**38%**	**23%**	**28%**	**30%**	

**Table 5:** Experiments with Gripper 4. On the left, the table with success rate for every object, in the last column, the average for every object is calculated, and at the end of the table, the average success rate for every environment is determined. On the right, the graphic representation of Table 5 is reported.

***Experiments with Gripper 4***					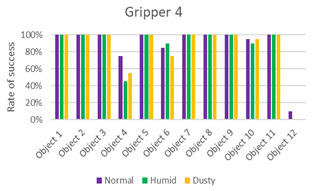
***Obj.***	**Normal**	**Humid**	**Dusty**	**Average**	
1	100%	100%	100%	100%	
2	100%	100%	100%	100%	
3	100%	100%	100%	100%	
4	75%	45%	55%	58%	
5	100%	100%	100%	100%	
6	85%	90%	75%	83%	
7	100%	100%	100%	100%	
8	100%	100%	100%	100%	
9	100%	100%	100%	100%	
10	95%	90%	95%	93%	
11	100%	100%	100%	100%	
12	10%	0%	0%	3%	
**Avg.**	**89%**	**85%**	**85%**	**86%**	

## Supplementary Materials

The following are available online at: https://youtu.be/fzxXVgQEElA, Supplementary materials Overview of the work and real tests.

## References

[1] Foumani M., Gunawan I., Smith-Miles K., Ibrahim M.Y. (2014). Notes on feasibility and optimality conditions of small-scale multifunction robotic cell scheduling problems with pickup restrictions. IEEE Trans. Ind. Inform..

[2] Tai K., El-Sayed A.R., Shahriari M., Biglarbegian M., Mahmud S. (2016). State of the art robotic grippers and applications. Robotics.

[3] Melchiorri C., Kaneko M. (2008). Springer Handbook of Robotics.

[4] Shintake J., Cacucciolo V., Floreano D., Shea H. (2018). Soft Robotic Grippers. Adv. Mater..

[5] Hirose S., Umetani Y. (1978). The development of soft gripper for the versatile robot hand. Mech. Mach. Theory.

[6] Dollar A.M., Howe R.D. (2006). A robust compliant grasper via shape deposition manufacturing. IEEE/ASME Trans. Mechatron..

[7] Manti M., Hassan T., Passetti G., D’Elia N., Laschi C., Cianchetti M. (2015). A bioinspired soft robotic gripper for adaptable and effective grasping. Soft Robot..

[8] Odhner L.U., Jentoft L.P., Claffee M.R., Corson N., Tenzer Y., Ma R.R., Buehler M., Kohout R., Howe R.D., Dollar A.M. (2014). A compliant, underactuated hand for robust manipulation. Int. J. Rob. Res..

[9] Liarokapis M., Dollar A.M. Post-contact, in-hand object motion compensation for compliant and underactuated hands. Proceedings of the 2016 25th IEEE International Symposium on Robot and Human Interactive Communication (RO-MAN).

[10] Stuart H.S., Wang S., Cutkosky M.R. (2018). Tunable contact conditions and grasp hydrodynamics using gentle fingertip suction. IEEE Trans. Robot..

[11] Mutlu R., Alici G., in het Panhuis M., Spinks G.M. (2016). 3D printed flexure hinges for soft monolithic prosthetic fingers. Soft Robot..

[12] Gafford J., Ding Y., Harris A., McKenna T., Polygerinos P., Holland D., Moser A., Walsh C. (2015). Shape deposition manufacturing of a soft, atraumatic, deployable surgical grasper. J. Med. Device..

[13] Liu C.-H., Huang G.-F., Chiu C.-H., Pai T.-Y. (2018). Topology synthesis and optimal design of an adaptive compliant gripper to maximize output displacement. J. Intell. Robot. Syst..

[14] Udupa G., Sreedharan P., Sai Dinesh P., Kim D. (2014). Asymmetric bellow flexible pneumatic actuator for miniature robotic soft gripper. J. Robot..

[15] Galloway K.C., Becker K.P., Phillips B., Kirby J., Licht S., Tchernov D., Wood R.J., Gruber D.F. (2016). Soft robotic grippers for biological sampling on deep reefs. Soft Robot..

[16] Hao Y., Gong Z., Xie Z., Guan S., Yang X., Ren Z., Wang T., Wen L. Universal soft pneumatic robotic gripper with variable effective length. Proceedings of the 2016 35th Chinese control conference (CCC).

[17] Guo J., Sun Y., Liang X., Low J.-H., Wong Y.-R., Tay V.S.-C., Yeow C.-H. Design and fabrication of a pneumatic soft robotic gripper for delicate surgical manipulation. Proceedings of the 2017 IEEE International Conference on Mechatronics and Automation (ICMA).

[18] Guo J., Elgeneidy K., Xiang C., Lohse N., Justham L., Rossiter J. (2018). Soft pneumatic grippers embedded with stretchable electroadhesion. Smart Mater. Struct..

[19] Li Y., Chen Y., Li Y. (2019). Pre-charged pneumatic soft gripper with closed-loop control. IEEE Robot. Autom. Lett..

[20] Mantriota G. (2007). Optimal grasp of vacuum grippers with multiple suction cups. Mech. Mach. Theory.

[21] Krahn J.M., Fabbro F., Menon C. (2017). A soft-touch gripper for grasping delicate objects. IEEE/ASME Trans. Mechatron..

[22] Brown E., Rodenberg N., Amend J., Mozeika A., Steltz E., Zakin M.R., Lipson H., Jaeger H.M. (2010). Universal robotic gripper based on the jamming of granular material. Proc. Natl. Acad. Sci. USA.

[23] Amend J., Cheng N., Fakhouri S., Culley B. (2016). Soft robotics commercialization: Jamming grippers from research to product. Soft Robot..

[24] Guo Z., Sun Z., Li X. Design and fabrication of pneumatic soft gripper. Proceedings of the ASME 2018 International Mechanical Engineering Congress and Exposition.

[25] Mizushima K., Oku T., Suzuki Y., Tsuji T., Watanabe T. Multi-fingered robotic hand based on hybrid mechanism of tendon-driven and jamming transition. Proceedings of the 2018 IEEE International Conference on Soft Robotics (RoboSoft).

[26] Fei Y., Wang J., Pang W. (2019). A novel fabric-based versatile and stiffness-tunable soft gripper integrating soft pneumatic fingers and wrist. Soft Robot..

[27] Nguyen P.H., Sridar S., Amatya S., Thalman C.M., Polygerinos P. Fabric-based soft grippers capable of selective distributed bending for assistance of daily living tasks. Proceedings of the 2019 2nd IEEE International Conference on Soft Robotics (RoboSoft).

[28] Nassour J., Hamker F. Enfolded textile actuator for soft wearable robots. Proceedings of the 2019 IEEE International Conference on Cyborg and Bionic Systems (CBS).

[29] Monkman G. (2003). Electroadhesive microgrippers. Ind. Robot An Int. J..

[30] Shintake J., Rosset S., Schubert B., Floreano D., Shea H. (2016). Versatile soft grippers with intrinsic electroadhesion based on multifunctional polymer actuators. Adv. Mater..

[31] Shea H.R., Jun S., Dario F. (2016). Soft Compliant Gripper for Safe Manipulation of Extremely Fragile Objects.

[32] Song S., Majidi C., Sitti M. Geckogripper: A soft, inflatable robotic gripper using gecko-inspired elastomer micro-fiber adhesives. Proceedings of the 2014 IEEE/RSJ International Conference on Intelligent Robots and Systems.

[33] Glick P., Suresh S.A., Ruffatto D., Cutkosky M., Tolley M.T., Parness A. (2018). A soft robotic gripper with gecko-inspired adhesive. IEEE Robot. Autom. Lett..

[34] Shintake J., Schubert B., Rosset S., Shea H., Floreano D. Variable stiffness actuator for soft robotics using dielectric elastomer and low-melting-point alloy. Proceedings of the 2015 IEEE/RSJ International Conference on Intelligent Robots and Systems (IROS).

[35] Xu L., Gu G. Bioinspired Venus flytrap: A dielectric elastomer actuated soft gripper. Proceedings of the 2017 24th International Conference on Mechatronics and Machine Vision in Practice (M2VIP).

[36] Guo J., Xiang C., Rossiter J. (2018). A soft and shape-adaptive electroadhesive composite gripper with proprioceptive and exteroceptive capabilities. Mater. Des..

[37] Pourazadi S., Bui H., Menon C. (2019). Investigation on a soft grasping gripper based on dielectric elastomer actuators. Smart Mater. Struct..

[38] Wang Z., Chathuranga D.S., Hirai S. 3D printed soft gripper for automatic lunch box packing. Proceedings of the 2016 IEEE International Conference on Robotics and Biomimetics (ROBIO).

[39] Marchese A.D., Katzschmann R.K., Rus D. (2015). A recipe for soft fluidic elastomer robots. Soft Robot..

[40] Pettersson A., Davis S., Gray J.O., Dodd T.J., Ohlsson T. (2010). Design of a magnetorheological robot gripper for handling of delicate food products with varying shapes. J. Food Eng..

[41] Hamid A.M.B., Makhdoomi M.R., Saleh T., Bhuiyan M. (2015). Development of a Shape Memory Alloy (SMA) Based Assistive Hand. Adv. Mater. Res..

[42] Kang L., Seo J.-T., Kim S.-H., Kim W.-J., Yi B.-J. (2019). Design and Implementation of a Multi-Function Gripper for Grasping General Objects. Appl. Sci..

[43] Soft Robotics Inc. https://www.softroboticsinc.com/.

[44] Empire Robotics. https://www.empirerobotics.com/products/.

[45] Festo MultiChoiceGripper. https://www.festo.com/group/en/cms/10221.htm.

[46] Recreus Recreus. https://recreus.com/en/.

